# Immersive Rehabilitation Therapy (MoveR) Improves Postural and Visuo-Attentional Skills in Children with ADHD: A Clinical Study

**DOI:** 10.3390/life16020257

**Published:** 2026-02-02

**Authors:** Simona Caldani, Ana Moscoso, Alexandre Michel, Eric Acquaviva, Charlotte Gibert, Florent Roger, Richard Delorme, Maria-Pia Bucci

**Affiliations:** 1 ICAR UMR 5191, CNRS, ENS de Lyon, Université Lyon 2, 69001 Lyon, France; simona.caldani@gmail.com (S.C.);; 2 Equipe InDev, NeuroDiderot, Robert Debré Hospital, Boulevard Sérurier, 75019 Paris, France; 3 Child and Adolescent Psychiatry Department, Robert Debré Hospital, APHP & Université Paris Cité, 75019 Paris, France; alexandre.michel@aphp.fr (A.M.); eric.acquaviva@aphp.fr (E.A.); richard.delorme@aphp.fr (R.D.); 4 Child and Adolescent Psychiatry Departement, Centre Hospitalier Universitaire, 76000 Rouen, France; char.gibert@hotmail.fr; 5 Paramedical Center Louis Guillet, 17000 La Rochelle, France; florent.roger.ortho@gmail.com; 6 Human Genetics and Cognitive Functions, Institut Pasteur, 75015 Paris, France

**Keywords:** ADHD, children, eye movements, posture, immersive training, cerebellum

## Abstract

**Background:** Motor as well as attentional skills are deficient in children with attention deficit hyperactivity disorder (ADHD). The aim of the present study was to explore whether a short immersive rehabilitation therapy could improve motor and visuo-attentional capabilities in children with ADHD. **Methods:** Forty children with ADHD participated in this study; IQ-, sex- and age-matched children were splitted in two groups (G1 and G2) of twenty. An unpredictable random sequence was used to allocate a child to group G1 (trained group) or G2 (control group). Oculomotor and postural performance for both groups of children were objectively assessed twice (before and after 16 min) by using an eye tracker and platform. Group G1 only underwent 16 min of immersive rehabilitation therapy, while the control group (G2) had 16 min of resting. The immersive therapy consisted of performing physical movement while training visual discrimination, attention and spatial orientation skills. **Results:** After 16 min, significant improvements in the fixation area (*p* = 0.008) and in the number of catch-up saccades during pursuit eye movements (*p* < 0.001), as well as a smaller postural instability index (PII) (*p* < 0.001), were observed for the trained group (G1) only. **Conclusions:** These findings suggest that children with ADHD could benefit from a short immersive therapy to improve both visual–attention and motor performances. This new immersive therapy is a useful tool allowing a better integration of both visual and motor sensory inputs via the cortico/cerebellar network. Follow-up studies on a larger number of children with ADHD will be necessary to explore the eventual possible persistence of such a training effect and imaging works will help to understand where such adaptive mechanisms take place.

## 1. Introduction

Attention deficit/hyperactivity disorder (ADHD) is one of the most common neurodevelopmental disorders in children, with a global prevalence estimated at around 5–8% [1,2]. Beyond inattention, hyperactivity and impulsivity, the disorder is also characterized by difficulties in controlling motor impulses [3].

For decades, the cerebellum has been identified as a central contributor to motor learning processes and coordination, but numerous studies have also highlighted its involvement in cognition, working memory, attention and emotion [4]. Recent work by Morgado et al. (2024) [5] reported significant differences between children with ADHD and controls in cerebellar regions associated with the attentional network and in the dorsal attention network, in line with earlier findings [6,7]. The acquisition of new motor and non-motor skills appears to rely on an adaptive control loop involving the cerebellum, which integrates complex sensory information [8].

Building on this framework, several studies have explored training-based interventions aimed at improving attention and cognitive functioning in children with ADHD. Both cognitive training programs and different neurofeedback protocols were extensively studied, but a comprehensive review of recent meta-analyses revealed significant limitations in the real-world clinical efficacy of prominent non-pharmacological ADHD interventions [9,10], yet simultaneously pointed toward a promising new direction involving the integration of motor tasks. Rigorous analysis of randomized controlled trials with “probably blinded” outcomes demonstrates that Computerized Cognitive Training (CCT), based on probably blinded reports, shows no effect on ADHD total symptoms or hyperactivity/impulsivity. While CCT does produce validated improvements in trained skills such as working memory, these gains do not translate into meaningful clinical effects on core ADHD symptoms; the only observed impact is a small, setting-specific improvement in inattention [9]. Similarly, meta-analyses of probably blinded reports show no significant improvement in ADHD total symptoms for neurofeedback, leading to the conclusion that it is not supported as a stand-alone treatment [10]. This therapeutic gap has prompted an alternative hypothesis: incorporating motor tasks into treatment regimens may enhance cognitive outcomes. Emerging evidence supports this view, with an exploratory study finding that children with ADHD who used motor primers while performing a semantic memory task had improved learning outcomes [11]. This finding is reinforced by multimodal rehabilitation studies; for instance, perceptual–motor exercises have been shown to decrease ADHD symptoms [12], while separate postural exercises have been found to significantly improve balance, suggesting “the presence of adaptive mechanisms in the sensory process and better cerebellar integration.” [13,14]. Therefore, the inclusion of motor tasks could present a promising avenue to overcome the constraints of purely cognitive training. The underlying rationale, directly supported by the postural improvement findings, is that such multimodal stimulation may facilitate better cerebellar integration of sensory inputs, potentially inducing synaptic adjustments in fronto-cerebellar networks [15,16] and leading to more robust and generalizable improvements in both attentional and motor skills for individuals with ADHD.

This study aimed to determine whether short immersive rehabilitation training (MoveR) could improve both visual attention and postural abilities in children with ADHD, as assessed using an eye tracker and a postural platform.

## 2. Methods

### 2.1. Subjects

Two groups (G1 and G2) of twenty children with ADHD were included in this study (see Table 1). Children were recruited at the Child and Adolescent Psychiatry Department of Robert Debré pediatric university hospital (Paris, France). They were diagnosed by experienced clinicians through clinical assessment following the DSM-5 criteria [3]. The severity and diversity of symptoms were assessed with the ADHD Rating Scale [17], and cognitive skills were explored using the Weschler scales. To be included in this study, children had to be diagnosed with ADHD, drug-naïve, aged between 6 and 12 years, and have all sub-scores of the intellectual quotient within the normal range (80–120). The allocation of a child to a specific group (trained group, G1, or control group, G2) was generated in an unpredictable random sequence; group G1 only benefited from the immersive rehabilitation therapy (MoveR).

**Figure 1:**
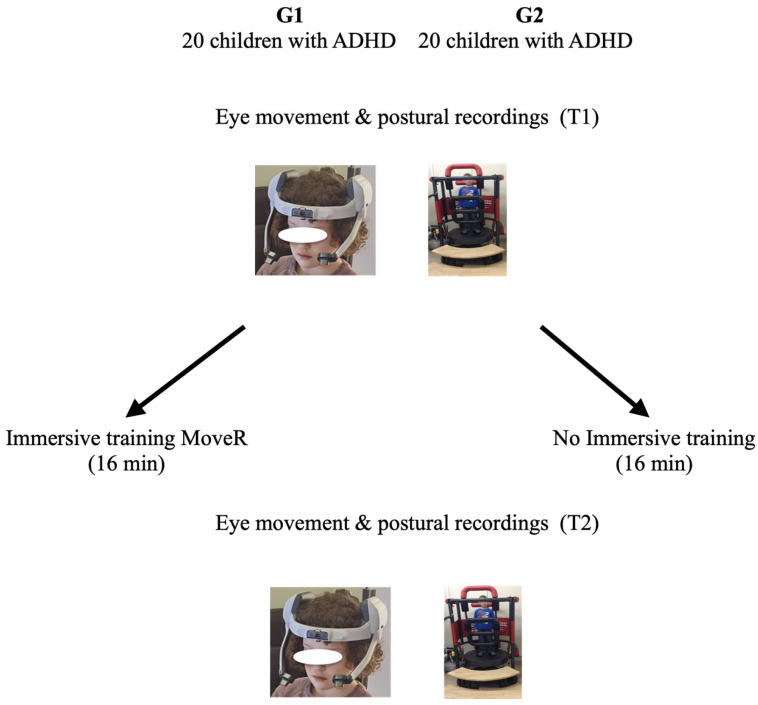
Description of the protocol used.

Eye recordings and body sway were assessed two times at T1 and T2. For group G2, these were before (T1) and after (T2) 16 min of rest (i.e., without immersive rehabilitation therapy); for group G1, these were before (T1) and after (T2) 16 min of immersive rehabilitation therapy. Figure 1 shows the trial design.

The investigation followed the principles of the Declaration of Helsinki and was approved by our Institutional Human Experimentation Committee (Comité de Protection des Personne CPP Île-de-France I (N° IDRCB: 2021-A00489-32). Written informed consent was obtained from the children’s parents after the experimental procedure was explained to them. Additionally, consent was obtained from the children, in a manner appropriate for their age and comprehension level.

### 2.2. Eye Movement Recordings

The Eya Eye Tracker (https://sierra-neurovision.com, accessed on 19 December 2025) was used to assess eye movements with a frequency of 120 Hz and an accuracy of 0.25 degrees. The child was in a dark room, seated on a chair 50 cm in front of a screen and had their head stabilized. At the beginning of the oculomotor tests, a calibration was performed. During this procedure, 9 points of 0.5 deg (diameter) were fixed on the screen and had to fixate on each point for 500 ms. A five-parameter polynomial function was used to extrapolate the calibration values [18]. After this procedure (calibration), three oculomotor exercises were shown: a fixation, a horizontal pursuit and an antisaccade task. The fixation task was similar to the one used in our previous study (see [19]); briefly, three black crosses were shown for 1 s on a screen. The child was asked to fixate on the center of the cross. The pursuit task consisted of following a target that was initially placed in the center of the screen and then moved horizontally at a velocity of 15 deg/s to one side until it reached the 20 deg location, where it reversed abruptly and moved to the opposite side. A total of nine trials were run and included in the analysis (see [20]). Antisaccades were also stimulated. The antisaccade task (originally introduced by Hallett 1978 [21]) consisted of presenting a visual stimulus on one side and asking the child to make a saccade to the opposite side. The central target was illuminated for a random period of 400, 600 or 800 ms. Simultaneously to the target extinction, a peripheral stimulus appeared and stayed on the screen for 300 ms. The child was invited to make a saccade to the opposite side of the stimulus as soon as possible. The duration of each task was kept short (lasting a couple of minutes), allowing an accurate evaluation of eye movement recordings.

### 2.3. Postural Recording

To evaluate postural ability, the Multitest Equilibre (www.framiral.fr, accessed on 19 December 2025) medical device was used [22]. The surface of the center of pressure displacement (CoP) was measured on an unstable platform under two different viewing conditions: eyes open (EO), fixating on a target (0.5°) at a distance of 250 cm (target projected on a screen in front of the subjects at their eye level), and eyes closed (EC) (see [23]). The displacement of the center of pressure (CoP) was sampled at 40 Hz and digitized with 16-bit precision. Postural recording was performed in a dark room while the child stood on the platform; the child was instructed to stay as still as possible with their arms along the side of the body and the feet position standardized on footprints (distance and angle between heels: 11 cm and 30°, respectively). Each postural condition was recorded over 30 s with 15 s of rest to avoid possible tiredness effects.

### 2.4. Immersive Rehabilitation Therapy (MoveR)

**Figure 2:**
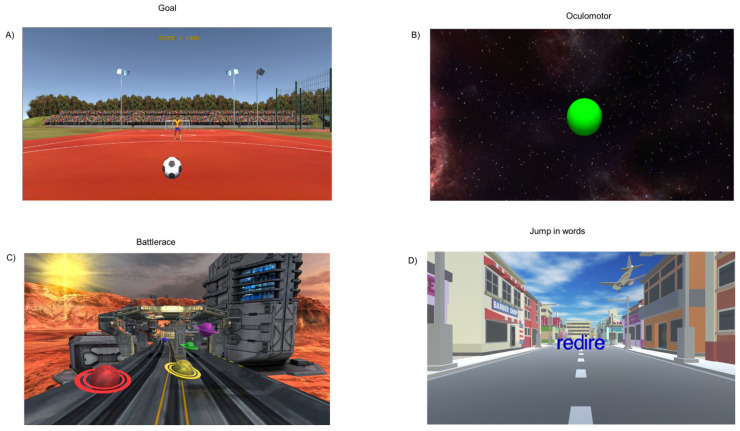
Examples of MoveR exercises: (**A**) Goal task; (**B**) Oculomotor task; (**C**) Battlerace task; (**D**) Jump in Words task.

MoveR is a new immersive rehabilitation training in which the child wears 3D glasses and different scenarios are controlled live by the experimenter (see [24]). The novelty of this type of rehabilitation is that the child performs a dual task by gaming. In detail, in this study, four different games were run, during which the child had to perform physical movements (the duration of each of them was 4 min). In the Goal task (Figure 2A), the child is in a football field and has to move their body to catch the ball kicked by the goalkeeper; in the Oculomotor task (Figure 2B), the child is in a street and has to fixate on or follow a target (by making saccades or pursuits through eye movements in different directions (horizontal, vertical and oblique)) and at the same time has to catch the target that could be in different positions with their hands; in Battlerace (Figure 2C), the child is walking on a road in a science fiction environment and has to catch the planets and avoid the obstacles that are coming from in front; and in Jump in Words (Figure 2D), the child is in front of a 3D word which gradually comes towards them, and has to jump on a specific letter of the word that is named by the experimenter.

All of these games have been developed in order to promote the multimodal approach to improving spatial and visual capabilities at the same time, combined with body movements to stimulate sensorial inputs.

### 2.5. Data Analysis

Eye tracking data analysis: For the fixation task, the gaze position of the dominant eye was recorded every 8.3 ms. This generated a point cloud for each eye, representing gaze location relative to the center of the fixation cross. An index of gaze stability was then calculated using the Bivariate Contour Ellipse Area (BCEA), with a confidence level set at 68% (see [19]). For the pursuit task, the number of catch-up saccades were measured. Catch-up saccades are saccades in the direction of the target, allowing the eyes to be brought closer to the target. For the antisaccades task, the mean error rate was measured (i.e., the number of saccades made in the target direction).

Postural control analysis: Postural control performance was evaluated measuring the postural instability index (PII) (see [25]). The PII enables evaluation of both postural oscillation and the energy demands of maintaining postural control, and it is a relevant parameter to report postural performance in children (see [25]). A large PII indicates instability. The data analyst was unaware of the children’s group allocation (G1 or G2).

### 2.6. Statistical Analysis

The Shapiro–Wilk test was used to test normality and the Levene test was used to verify homogeneity of variance. The Shapiro–Wilk test and Levene test showed *p* ≥ 0.05 for all variables tested; consequently, the Student *t*-test and ANOVA were applied. More precisely, we performed a Student *t*-test between the two groups of children (G1 and G2) to compare the clinical characteristics of children. A repeated-measures ANOVA was applied to the fixation area surface and catch-up saccades during pursuit, considering group (G1 and G2) as a between-subjects factor and the two time points (T1, T2) as within-subjects factors. A repeated-measures ANOVA was carried out to evaluate the postural instability index, considering group (G1 vs. G2) as a between-subjects factor and time (T1, T2) and visual condition (EO, EC) as within-subjects factors. We used JASP(Version 0.19.3) software for the analysis. The Bonferroni test was used for post hoc comparisons. The confidence interval was set at 95% and a 0.05 level of significance was adopted throughout the data analysis.

## 3. Results

In Table 1, clinical characteristics of the children are shown. The *t*-test failed to show any significant group effect for age, sex, ADHD-RS scores and Wechsler-scale (WISC-V) scores.

**Figure 3:**
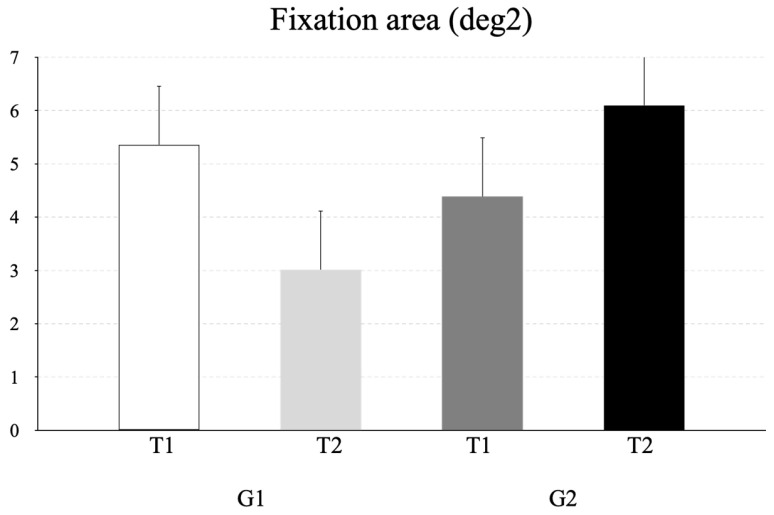
Mean and standard deviation of the fixation area (deg^2^) recorded during fixation task at T1 and T2 for G1 and G2.

Figure 3 illustrates the mean fixation area (deg^2^) measured at T1 and T2 for the two groups of children (G1 and G2). The repeated-measures ANOVA revealed a significant time × group interaction (F_(1,38)_ = 17.94, *p* = 0.001, η^2^ = 0.04). Bonferroni post hoc tests indicated that the fixation area was significantly smaller in G1 at T1 compared to T2. The Bonferroni post hoc test revealed that the fixation area was significantly smaller in group G1 at T1 with respect to T2 (*p* = 0.008, CI = 0.46 and 4.24 for lower and upper bounds, respectively).

**Figure 4:**
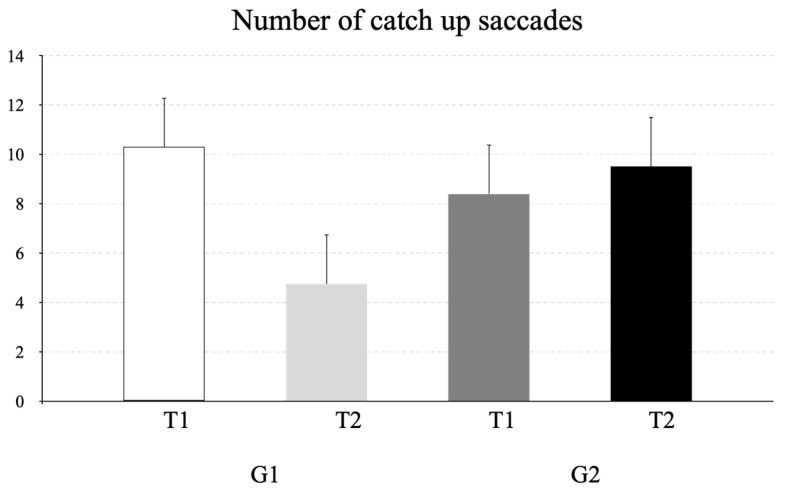
Mean number and standard deviation of catch-up saccades made in the pursuit task at T1 and T2 for G1 and G2.

The mean number of catch-up saccades observed during pursuits is shown in Figure 4. ANOVA reported a significant interaction time x group effect (F_(1,38)_ = 26.07, *p* < 0.001, η^2^ = 0.08). Post hoc analysis showed that G1 exhibited significantly fewer catch-up saccades at T2 than at T1 (*p* < 0.001, CI = 2.98 and 8.12 for lower and upper bounds, respectively).

**Figure 5:**
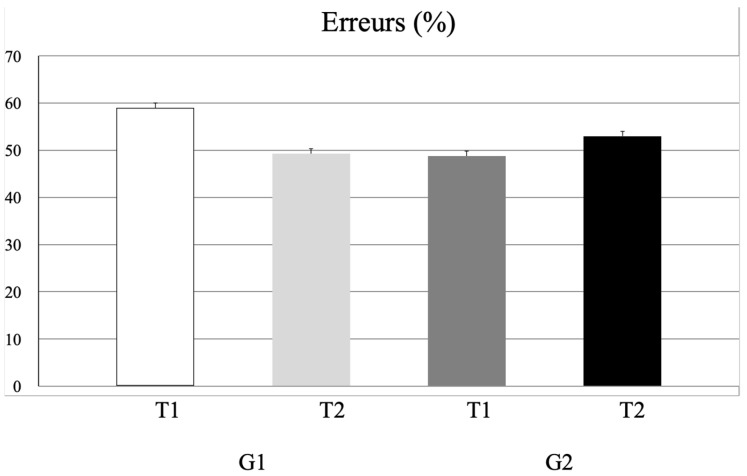
Mean and standard deviation of the errors (%) measured in the antisaccade task at T1 and T2 for G1 and G2.

ANOVA run on the number of antisaccade errors (see Figure 5) showed a significant interaction time x group effect y (F_(1,38)_ = 6.71, *p* < 0.01, η^2^ = 0.03); however, the Bonferroni test failed to show any significant difference (*p* = 0.89).

**Figure 6:**
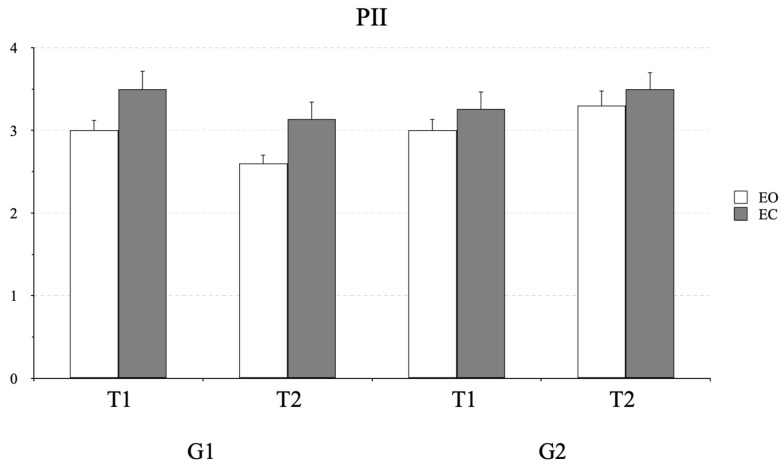
Mean and standard deviation of the postural instability indices (PIIs) assessed during postural tasks at T1 and T2 for G1 and G2 under eyes open (EO) and eyes closed (EC) conditions.

**Postural control.** Figure 6 shows the values of the postural instability index (PII) measured in G1 and G2 before and after 16 min of MoveR training. ANOVA indicated a significant interaction of time × group (F_(1,38)_ = 57.05, *p* < 0.001, η^2^ = 0.10). The PII significantly decreased from T1 to T2 (*p* < 0.001, CI = 0.33 and 0.92 for lower and upper bounds, respectively) in the G1 group. ANOVA also showed a significant effect of vision condition: under the EC condition, the PII was significantly higher (F_(1,38)_ = 18.21, *p* < 0.001, η^2^ = 0.08).

## 4. Discussion

The present study explored cognitive and motor benefits of short immersive rehabilitation training in children with ADHD. Eye movements (fixation, pursuit and antisaccades) as well as body sway have been recorded. We reported improved fixation and pursuit tasks as well as motor skills only in the group of children who received training.

Notably, both smooth pursuit and visual fixation rely on inhibitory control mechanisms to maintain gaze on a moving or stationary target, suppressing involuntary saccades [26]. In primates, single-cell recordings have revealed that a visual fixation task engages a specific neural system involving the frontal eye fields [27], the posterior parietal cortex [28,29] and brainstem formations [30]. Concerning pursuit movements, ref. [31] highlighted the involvement of the motion-sensitive area V5, frontal and parietal eye fields, supplementary eye fields, cerebellar vermis, angular gyrus and parieto-insular vestibular cortex. In line with these findings, we could suggest that this type of immersive rehabilitation training engaged an improvement in these oculomotor and attentional control networks, even if neuroimaging studies are necessary to confirm such hypotheses.

Regarding the antisaccade task, no significant improvements were observed following the training. This lack of effect may be attributed to the specific cognitive demands involved in performing antisaccades. Indeed, this task requires two distinct neurophysiological processes: (1) the inhibition of a reflexive saccade toward a suddenly appearing stimulus and (2) the voluntary initiation of a saccade in the opposite direction. Previous studies have shown that the dorsolateral prefrontal cortex plays a critical role in inhibitory control and error monitoring during antisaccades, whereas the frontal eye fields are more involved in the modulation of saccade latency [32]. Considering what has been observed, it is plausible to suggest that the type of shorter immersive training implemented here was not sufficient to induce measurable changes in the higher-order inhibitory mechanisms required for antisaccade performance. It is possible that longer or more targeted cognitive motor training may be necessary to impact these complex control processes. Ongoing studies are testing such hypotheses.

Concerning posture, an fMRI study conducted by Kim et al. [33] found that children with ADHD exhibit impaired postural regulation, possibly due to reduced connectivity in the premotor cortex. The cerebellum is also essential for combining sensory information from visual, vestibular and somatosensory systems involved in maintaining posture [34].

In the literature, it has been reported that physical exercise, increasing regional cerebral blood flow, could improve cognition [35]. Several studies in fact reported that physical activity interventions improved working memory and executive functioning and prefrontal cortex activity [36,37,38,39]. More recently, Wick et al. [40] explored the relation between physical activities and attention in 61 healthy children (mean age 4.5 ± 0.6 years) to define specific physical fitness components responsible for attention fluctuation. These authors reported that coordination during physical fitness was related to attention in preschool children. The study by Cerrillo-Urbina et al. [41] reviewed several studies and concluded that aerobic exercise programs significantly enhanced attention; reduced hyperactivity and impulsivity; and improved anxiety levels, executive function and social behavior in children with ADHD. These benefits were probably attributable to modulation of brain structures involved in cognitive control, such as the prefrontal and cingulate cortices, brainstem and cerebellum. Supporting this, Moradi et al. [13] found that seven weeks of balance training improved postural control in children with ADHD, potentially through adaptive sensory processing and enhanced cerebellar integration. Similarly, Shams et al. [42] emphasized the importance of attention in postural control, showing that targeted attention training can improve postural stability in this population.

Considering these findings, we hypothesize that this novel rehabilitation training—through multimodal stimulation—may foster improved cerebellar integration of sensory inputs and, consequently, may enhance both attentional and motor abilities in children with ADHD. Note however that such a hypothesis needs to be confirmed by neuroimaging studies.

## 5. Conclusions

In the present study, we reported that children with ADHD benefited from short immersive rehabilitation training. Specifically, we observed improvements in fixation and pursuit eye movements, as well as in postural control. These enhancements may reflect a more efficient use of attentional resources during visual and postural tasks, potentially leading to better cerebellar integration. However, no significant changes were observed in antisaccade performance, suggesting that higher-order inhibitory processes may require longer or more targeted training. Future research may investigate the persistence of these effects and their transfer to other cognitive domains.

## 6. Limitations

In this study, there are some limitations. First, we only measure the immediate effect of short immersive rehabilitation training, so it is not known if this training’s effects could persist after the exercise program. Further follow-up studies are required to confirm the persistence of these improvements over time in a larger sample of children. Second, to directly define the underlying neural mechanism, it is necessary to conduct neuroimaging measures. It will also be interesting to test the proposed intervention over the longer term, for instance, after several training sessions. The control group is not an active control; clinical transfer or functional impact is not evaluated. Moreover, in the future it could be more exhaustive to propose different types of cognitive training associated with motor tasks to examine the effect of each intervention on ADHD pathology.

## Figures and Tables

**Table 1:** Characteristics of the children tested and enrolled in this study (trained and control groups, G1 and G2, respectively).

	G1 N = 20 Children with ADHD	G2 N = 20 Children with ADHD	*p* Values
Age (year)	10 ± 1	10.9 ± 1	0.10
Sex (F/M)	3/17	4/16	0.22
**ADHD-RS Scores:**			
ADHD-RS Total Score	38 ± 2	39 ± 1	0.28
ADHD-RS Inattention Sub-Score	18 ± 1	19 ± 1	0.17
ADHD-RS Hyperactivity/Impulsivity Sub-Score	19 ± 2	20 ± 2	0.15
**WISC-V (Wechsler Intelligence Scale for Children) Scores:**			
Verbal Comprehension Index	98 ± 3	97 ± 6	0.11
Visual Spatial Index	91 ± 5	93 ± 3	0.23
Working Memory Index	96 ± 4	97 ± 4	0.27
Fluid Reasoning Index	100 ± 4	101 ± 3	0.21
Processing Speed Index	97 ± 5	96 ± 6	0.19

## Data Availability

The datasets generated and/or analyzed during the current study are available from the author on reasonable request. The data is not publicly available due to ethical restrictions.
